# Exposure to household secondhand smoke among adolescents in Kuwait: Results from two school-based cross-sectional studies

**DOI:** 10.18332/tid/119116

**Published:** 2020-04-07

**Authors:** Ali H. Ziyab, Mohammad Almari, Abdullah Al-Taiar

**Affiliations:** 1 Department of Community Medicine and Behavioral Sciences, Faculty of Medicine, Kuwait University, Kuwait City, Kuwait; 2 Department of Health Policy and Management, Faculty of Public Health, Kuwait University, Kuwait City, Kuwait; 3 School of Community and Environmental Health, College of Health Sciences, Old Dominion University, Norfolk, United States

**Keywords:** secondhand smoke, adolescents, Kuwait, epidemiology

## Abstract

**INTRODUCTION:**

Detrimental effects of secondhand smoke (SHS) exposure are well established; however, data on SHS exposure among adolescents in Kuwait are lacking. Hence, this study sought to estimate the prevalence of household SHS exposure among two samples of adolescents in Kuwait and assess its variation by socioeconomic status and parental education level.

**METHODS:**

Data from two large school-based cross-sectional studies were analyzed. Adolescents attending public middle (n=3864; aged 11–14 years) and high (n=1959; aged 14–19 years) schools throughout Kuwait were enrolled in 2016-2017, and parental self-reported household SHS exposure was ascertained. Associations were assessed using Poisson regression with robust variance estimation, and adjusted prevalence ratios (APRs) and 95% confidence intervals (CIs) were estimated.

**RESULTS:**

Overall, 45.8% (1755/3836; 95% CI: 44.2–47.3%) of the enrolled middle school students and 51.6% (998/1936; 95% CI: 49.3–53.8%) of the enrolled high school students were exposed to household SHS. Among middle and high school students, the prevalence of household SHS exposure increased as maternal/paternal education level and family income decreased. Among middle school students, paternal educational attainment of middle school or less compared to bachelor’s degree or higher was associated with 1.60 times (95% CI: 1.44–1.79) higher household SHS exposure. Similarly, in the sample of middle school students, the prevalence of household SHS exposure significantly increased from 35.8% among children from families reporting the highest household income to 50.5% among children from families with the lowest reported household income (p-trend<0.001).

**CONCLUSIONS:**

Household SHS exposure is substantially high among adolescents in Kuwait. Enrolled adolescents from families with low socioeconomic status or with low parental education level have the highest household SHS exposure. These findings highlight the need for national comprehensive tobacco control policies and increasing parental awareness of the impact of SHS exposure on children.

## INTRODUCTION

Tobacco smoking, a preventable cause of morbidity and mortality, remains a major global public health challenge. No less, exposure to secondhand smoke (SHS), also referred to as passive or environmental tobacco smoke, is associated with detrimental health effects for children and non-smoker adults. It is becoming an indisputable fact that there is no safe level of exposure to SHS^1^ and that SHS exposure during childhood and adulthood has been linked to mortality in adults who never smoke^2^. Children exposed to SHS have a higher risk of sudden infant death syndrome, acute respiratory infection, severe asthma, and slow lung growth^1^. Moreover, accumulated evidence suggests that SHS exposure is associated with cardiovascular consequences in children^3^. Similarly, adults exposed to SHS have a higher risk of lung cancer and other cancers in addition to adverse effects on the cardiovascular system including arterial stiffness, atherosclerosis, and endothelial dysfunction^1,4^.

Global estimates from 2004 showed that 40% of children, 35% of adult female non-smokers, and 33% of adult male non-smokers were exposed to SHS^5^. Among adolescents living in low- and middle-income countries, the overall prevalence of SHS exposure was estimated to be 55.9%, with the lowest estimate of 16.4% reported in Tajikistan and the highest estimate of 85.4% reported in Indonesia^6^. Decreasing trends in SHS exposure among children have been reported in several countries, including Germany^7^, Australia^8^, Denmark^9^, the United Kingdom^10-12^, and the United States^13^. Nevertheless, socioeconomic disparities in the prevalence of SHS exposure among children exist. For instance, exposure to SHS is highest among children of parents with lower education attainment^14-16^. Similarly, family income has been shown to be inversely associated with childhood exposure to SHS^16-18^. Such socioeconomic disparities seem to persist over time^7,8,10^.

Analysis of the burden of disease from SHS exposure showed that 61% of total SHS-attributable disability-adjusted life-years were in children^5^. Hence, developing, implementing and enforcing comprehensive smoke-free laws in all indoor workplaces and public places, as well as smoke-free home and vehicle rules, will probably reduce the burden attributed to SHS exposure among non-smoker adults and children/adolescents. Although epidemiologic characterization of SHS exposure in most developed countries is thorough, some countries still lack such empirical knowledge. Hence, to inform public health officials and policymakers, this study aimed to report, for the first time, the magnitude of household SHS exposure among samples of middle and high school students in Kuwait and to describe disparities in household SHS exposure according to parental education and socioeconomic status.

## METHODS

### Study setting, design and population

Data from two large school-based cross-sectional studies were analyzed and reported in this investigation. The first study was a cross-sectional study that enrolled schoolchildren (n=3864) attending public middle schools across all governorates of Kuwait, which included children aged between 11 and 14 years. The schoolchildren were enrolled in the study during the 2016–2017 school year (September 2016 to May 2017) and the first semester of the 2017–2018 school year (September to December 2017). A stratified two-stage cluster sampling method was used to select a representative study sample of schoolchildren from a random sample of middle schools across Kuwait. The sampling methodology is described in detail elsewhere^19^. In the current report, we analyzed data from 3836 participants with information on household SHS exposure (i.e. excluding 28 participants with missing information). Parents provided written informed consent to enrol their children in the study and completed study questionnaires. Ethical approval was obtained from the Standing Committee for Coordination of Health and Medical Research, Ministry of Health, Kuwait (No. 2016/451).

The second study was a cross-sectional study that enrolled high school students (n=1959) attending public schools across Kuwait, which typically include students aged between 14 and 19 years. Recruitment of study participants occurred during the first semester of the 2017–2018 school year (September to December 2017). To ensure a representative study sample, a cluster random sampling method was used in selecting schools and students, as detailed by Almari et al.^20^. In this analysis, data from 1936 participants with information on household SHS exposure were used (i.e. excluding 23 participants with missing information). Upon obtaining parental written informed consent, self-administered questionnaires were completed by parents and students. This study was approved by the Health Sciences Center Ethical Committee at Kuwait University (No. VDR/EC/3067). It is worth noting that school enrollment is very high in Kuwait for both females and males and that data from both studies can be extrapolated to all adolescents in the country.

### Ascertainment of SHS exposure and covariates

In both studies, exposure to household SHS was ascertained by an affirmative response by parents to the question: ‘Does anyone smoke cigarettes or waterpipe inside the house?’. This approach allowed us to estimate the point prevalence of household SHS exposure. Age was categorized as: ≤11, 12, 13, and ≥14 years in the middle school students, with age ≤11 years being the reference group. Moreover, among high school students, age was reported as: ≤15, 16, and ≥17 years, with age ≤15 years being the reference group. Information on maternal and paternal level of education was obtained in both studies with the following options: middle school or less, high school, diploma (a two-year associate degree post high school), and bachelor’s degree or higher as the reference group. Total monthly family income in Kuwaiti Dinar (1 KWD about 3.21 US$) was reported by parents in both studies as: <1000, 1000–1500, 1501–2000, 2001–2500, 2501–3000, and >3000 as the reference group. Information on cigarette self-smoking was sought from the enrolled high school students by asking the following question: ‘Have you smoked at least one combustible cigarette in the past 30 days?’. This approached allowed us to determine current cigarette smoking status.

### Statistical analysis

All statistical analyses were conducted using SAS 9.4 (SAS Institute, Cary, North Carolina, USA). The statistical significance level was set to α=0.05. Prevalence of household SHS exposure was determined in the total study samples and across categories of different variables. Prevalence ratios (PRs) and 95% confidence intervals (CIs) that assessed associations between different covariates and SHS exposure were estimated by applying a modified Poisson regression with robust variance estimation using the GENMOD procedure in SAS 9.4^21^.

## RESULTS

Table 1 shows characteristics of the two analyzed study samples. In total, 3864 middle school students were enrolled, of which 3836 (2154 girls and 1682 boys) participants were analyzed in this report (i.e. excluding 28 participants with missing information on household SHS exposure; Table 1). The median (5th, 95th percentile) age of the middle school study participants was 12.0 (11.0, 14.0) years. Majority of the enrolled middle school children’s mothers (42.7%, 1626/3806) and fathers (35.7%, 1353/3786) reported having a bachelor’s degree or higher. The most commonly reported (28.5%, 978/3426) total monthly household income was between 1000 and 1500 KWD among middle school participants. On the other hand, 1959 high school students were enrolled, of which 1936 (1049 girls and 887 boys) were analyzed in this report (i.e. excluding 23 participants with missing information on household SHS exposure). The majority of the enrolled high school students were aged between 14 years (5th percentile) and 18 years (95th percentile), with a median age of 16 years. Majority of mothers (39.5%, 762/1928) and fathers (39.2%, 753/1921) of the enrolled high school students reported having an education level of bachelor’s degree or higher. Among high school participants, the most commonly reported (26.0%, 275/1057) total monthly household income was between 1000 and 1500 KWD.

**Table 1:** Characteristics of the two analyzed study samples of Kuwait, 2016–2017

*Characteristics Middle*	*School students (n=3836) % (n)*	*High School students (n=1936) % (n)*
**Adolescent gender**		
Female	56.2 (2154)	54.2 (1049)
Male	43.8 (1682)	45.8 (887)
Missing, (n)	(0)	(0)
**Adolescent age group** (years)		
≤11^a^	27.6 (1059)	–
12	30.2 (1159)	–
13	24.9 (956)	–
≥14^b^	17.3 (662)	–
Missing, (n)	(0)	–
≤15^c^	–	35.1 (676)
16	–	29.8 (575)
≥17^d^	–	35.1 (675)
Missing, (n)	–	(10)
**Maternal education**		
Middle school or less	14.5 (553)	16.2 (312)
High school	18.5 (703)	26.7 (514)
Diploma^e^	24.3 (924)	17.6 (340)
Bachelor’s degree or higher	42.7 (1626)	39.5 (762)
Missing, (n)	(30)	(8)
**Paternal education**		
Middle school or less	18.1 (684)	16.5 (316)
High school	23.1 (876)	27.8 (535)
Diploma^e^	23.1 (873)	16.5 (317)
Bachelor’s degree or higher	35.7 (1353)	39.2 (753)
Missing, (n)	(50)	(15)
**Monthly household income** (KWD)		
<1000	14.2 (487)	16.4 (173)
1000–1500	28.5 (978)	26.0 (275)
1501–2000	16.9 (579)	17.4 (184)
2001–2500	15.1 (516)	11.8 (125)
2501–3000	12.5 (428)	14.6 (154)
>3000	12.8 (438)	13.8 (146)
Missing, (n)	(410)	(879)

KWD: 1 Kuwaiti Dinar about 3.21 US$.

a The youngest age reported was 9 years.

b The oldest age reported was 17 years.

c The youngest age reported was 12 years.

d The oldest age reported was 20 years.

e Refers to a two-year associate degree post high school.

Overall, 45.8% (1755/3836; 95% CI: 44.2–47.3) of the enrolled middle school students and 51.6% (998/1936; 95% CI: 49.3–53.8) of the enrolled high school students were exposed to household SHS (Table 2). Among the enrolled middle and high school students, exposure to household SHS was similar across gender. Exposure to household SHS increased as age increased. For example, among the sample of high school students, 48.1% of those aged ≤15 years were exposed compared to 56.3% of those aged ≥17 years. Among the sample of middle and high school students, household SHS exposure prevalence showed significant increasing trends as maternal/paternal education level and monthly household income decreased. For instance, among middle school students who participated in the study, paternal educational attainment of middle school or less compared to bachelor’s degree or higher was associated with 1.60 times (95% CI: 1.44–1.79) higher household SHS exposure. In regard to monthly household income, among the study sample, 50.5% of middle school students from families reporting income of less than 1000 KWD a month were exposed to household SHS compared to 35.8% of middle school students who reported the highest monthly family income of more than 3000 KWD.

**Table 2:** Prevalence estimates of household secondhand smoke (SHS) exposure in the total population and according to personal and familial characteristics: results from the two analyzed studies

*Characteristics*	*Middle School students*		*High School students*	

	*SHS exposure % (n/total)*	APR* (95% CI)	*SHS exposure % (n/total)*	APR* (95% CI)
**Total**	45.8 (1755/3836)	–	51.6 (998/1936)	–
**Adolescent gender**				
Female	46.3 (998/2154)	1.00 (Ref.)	52.5 (551/1049)	1.00 (Ref.)
Male	45.0 (757/1682)	0.99 (0.92–1.07)	50.4 (447/887)	0.96 (0.88–1.04)
p-value	0.413		0.349	
**Adolescent age group** (years)				
≤11^a^	42.5 (450/1059)	1.00 (Ref.)	–	–
12	47.5 (550/1159)	1.09 (0.99–1.20)	–	–
13	46.6 (445/956)	1.07 (0.97–1.18)	–	–
≥14^b^	46.8 (310/662)	1.05 (0.95–1.18)	–	–
≤15^c^	–	–	48.1 (325/676)	1.00 (Ref.)
16	–	–	50.3 (289/575)	1.02 (0.91–1.13)
≥17 years^d^	–	–	56.3 (380/675)	1.11 (1.01–1.23)
p-value	0.092		0.008	
**Maternal education**				
Middle school or less	50.6 (280/553)	1.07 (0.97–1.17)	55.8 (174/312)	1.08 (0.95–1.24)
High school	52.8 (371/703)	1.13 (1.02–1.25)	56.0 (288/514)	1.11 (0.99–1.24)
Diploma^e^	46.7 (431/924)	1.03 (0.92–1.16)	54.1 (184/340)	1.14 (1.01–1.29)
Bachelor’s degree or higher	40.5 (659/1626)	1.00 (Ref.)	45.4 (346/762)	1.00 (Ref.)
p-value	<0.001		<0.001	
**Paternal education**				
Middle school or less	59.1 (404/684)	1.60 (1.44–1.79)	59.8 (189/316)	1.28 (1.13–1.46)
High school	53.0 (464/876)	1.45 (1.30–1.61)	57.6 (308/535)	1.25 (1.12–1.40)
Diploma^e^	47.0 (410/873)	1.31 (1.17–1.46)	51.4 (163/317)	1.14 (1.00–1.31)
Bachelor’s degree or higher	33.5 (453/1353)	1.00 (Ref.)	44.0 (331/753)	1.00 (Ref.)
p-value	<0.001		<0.001	
**Monthly household income**				
<1000	50.5 (246/487)	1.16 (0.98–1.37)	57.8 (100/173)	1.53 (1.20–1.95)
1000–1500	51.1 (500/978)	1.15 (0.99–1.34)	53.2 (146/275)	1.29 (1.02–1.62)
1501–2000	48.7 (282/579)	1.14 (0.98–1.34)	52.2 (96/184)	1.23 (0.96–1.56)
2001–2500	44.4 (229/516)	1.12 (0.96–1.31)	52.8 (66/125)	1.28 (0.99–1.65)
2501–3000	38.6 (165/428)	1.01 (0.85–1.20)	53.9 (83/154)	1.33 (1.05–1.69)
>3000	35.8 (157/438)	1.00 (Ref.)	40.4 (59/146)	1.00 (Ref.)
p-value	<0.001		0.013	

KWD: 1 Kuwaiti Dinar about 3.21 US$. SHS: secondhand smoke. CI: confidence interval.

* APR: adjusted prevalence ratio, simultaneously adjusted for all variables shown in the Table.

a The youngest age reported was 9 years.

b The oldest age reported was 17 years.

c The youngest age reported was 12 years.

d The oldest age reported was 20 years.

e Refers to a two-year associate degree post high school.

Additional analysis on the role of self-smoking on SHS exposure prevalence was conducted among the sample of high school students. The prevalence of current cigarette self-smoking among the enrolled high school students was estimated to be 13.7% (264/1925; data not shown). Among those who are not current cigarette smokers, the prevalence of household SHS exposure was 49.4% (821/1661), whereas among those who are current cigarette smokers, the prevalence of SHS exposure was 65.5% (173/264).

## DISCUSSION

This study sought to estimate the prevalence of household SHS exposure among adolescents (samples of middle and high school students) in Kuwait and explore disparities in household SHS exposure according to parental education and socioeconomic status. The findings of this study demonstrated that nearly half of adolescents enrolled in the analyzed cross-sectional studies in Kuwait are exposed to household SHS (45.8% of middle school students and 51.6% of high school students). Estimates of SHS exposure in this report are higher than the estimate from the Global Youth Tobacco Survey that found 30.4% of 356414 never-smoking adolescents in 168 countries to be exposed to household SHS^22^. Similarly, our estimates of SHS exposure exceeded a prior global estimate of 40% among children^5^. However, our estimates are slightly lower than another global estimate among adolescents living in low- and middle-income countries of 55.9%^6^. At the regional level, household SHS exposure among a sample of middle and high school students in Saudi Arabia was similar to our findings at 49.3%^23^. Moreover, our results demonstrated that enrolled adolescents of mothers and fathers with low educational attainment and those from families with low income were the most vulnerable to household SHS exposure. Such observations are consistent with previous investigations that showed low socioeconomic status and low parental education are associated with higher SHS exposure among children^24,25^.

Further analysis among the enrolled high school students showed that SHS exposure was higher among adolescents who reported current cigarette smoking compared to those who do not currently smoke cigarettes (65.5% vs 49.4%). Such an observation is in agreement with existing literature^26^. Moreover, this observation indicates that a significant proportion (49.4%) of non-smoking high school students in Kuwait are being exposed to household SHS. Such a result is comparable to results from 25 African countries that showed around 45% of never-smoking adolescents in Africa are exposed to SHS^27^. However, our estimate of 49.4% SHS exposure among non-smoking adolescents is higher than an estimate of 32.0% among non-smoking US adolescents aged 12– 19 years, in 2014^13^. Overall, these results are alarming and indicate that non-smoking adolescents in Kuwait are being exposed to high rates of household SHS.

The detrimental effects of SHS exposure on health and its predisposing effects on never-smoking adolescents to initiate smoking are demonstrated in the literature^28,29^. Moreover, emerging evidence suggesting that SHS exposure is a risk factor for delayed neurodevelopment and cognitive impairment in children further highlights the adverse effects of SHS exposure among children^30^. This report has documented that a substantial proportion of adolescents in Kuwait who participated in the analyzed studies are exposed to household SHS. Therefore, local public health policies should be developed to address this issue. Efforts should be made to increase parental awareness of the detrimental impact of SHS exposure on their children and be encouraged to ensure smoke-free homes and cars. Although laws banning smoking in public places have been enforced at the national level, there is clearly a need for policies and strategies to tackle smoking at the household level. To ultimately reduce SHS exposure, reducing smoking in the community should be addressed first.

### Strengths and limitations

A major strength of the current work is the representative and large samples of middle and high school students, which increases the external validity of our findings. A potential limitation is the possibility of underreporting of household SHS exposure by parents, which has been demonstrated by previous studies^18,31^. Another limitation to our study is the lack of confirmatory information on the extent of ‘inside’ the house exposure. For instance, smokers might choose to smoke in a separate room in the house or during the time when the children are not in the house. Hence, such practices might affect the true prevalence of SHS exposure.

Moreover, future studies should objectively assess household smoking-related air pollution and children’s urine cotinine to supplement and validate the self-reported SHS exposure. Current self-smoking status was assessed in the sample of high school students and showed that SHS exposure differs according to self-smoking status. The lack of information on self-smoking status in the sample of middle school students is a further limitation to our analysis. Moreover, not having information on the specific type of smoke (i.e. smoke from cigarettes, waterpipe etc.) that children are exposed to is a further limitation.

## CONCLUSIONS

A large proportion of participating adolescents in Kuwait are exposed to household SHS, which highlights the need for developing and implementing a comprehensive strategy to control tobacco consumption at the national level. Such a strategy will eventually reduce SHS exposure in the vulnerable youth population, particularly in disadvantaged families.
